# Topological hyperbolic metamaterials

**DOI:** 10.1515/nanoph-2023-0768

**Published:** 2024-02-21

**Authors:** Zhitong Li, Qing Gu

**Affiliations:** State Key Laboratory of Information Photonics and Optical Communications, School of Science, Beijing University of Posts and Telecommunications, Beijing 100876, China; Department of Electrical and Computer Engineering, North Carolina State University, Raleigh, NC 27695, USA; Department of Physics, North Carolina State University, Raleigh, NC 27695, USA

**Keywords:** hyperbolic dispersion, topological transition, loss compensation, all-dielectric hyperbolic metamaterial, twistronics, topological edge state

## Abstract

Hyperbolic metamaterial (HMM) is a unique type of anisotropic material that can exhibit metal and dielectric properties at the same time. This unique characteristic results in it having unbounded isofrequency surface contours, leading to exotic phenomena such as spontaneous emission enhancement and applications such as super-resolution imaging. However, at optical frequencies, HMM must be artificially engineered and always requires a metal constituent, whose intrinsic loss significantly limits the experimentally accessible wave vector values, thus negatively impacting the performance of these applications. The need to reduce loss in HMM stimulated the development of the second-generation HMM, termed active HMM, where gain materials are utilized to compensate for metal’s intrinsic loss. With the advent of topological photonics that allows robust light transportation immune to disorders and defects, research on HMM also entered the topological regime. Tremendous efforts have been dedicated to exploring the topological transition from elliptical to hyperbolic dispersion and topologically protected edge states in HMM, which also prompted the invention of lossless HMM formed by all-dielectric material. Furthermore, emerging twistronics can also provide a route to manipulate topological transitions in HMMs. In this review, we survey recent progress in topological effects in HMMs and provide prospects on possible future research directions.

## 1 Introduction

In 2000, John Pendry theoretically eluded the possibility of creating a perfect lens with materials that have negative indices of refraction [1]. The proposed perfect lens possesses super-resolution, with which evanescent waves can be resolved to overcome the diffraction limit of the light. Since then, many efforts have been put toward experimentally realizing negative index materials at optical frequencies [2], [3], [4]. Incidentally, in the same year, Smith et al. experimentally demonstrated a composite medium, consisting of periodic metallic split ring resonators and continuous wires, that exhibits negative permittivity and permeability simultaneously at microwave frequencies [5]. This type of material is called “left-handed” medium, because the well-known Doppler effect, Cherenkov radiation, and Snell’s law are all inverted in such material. Subsequently, in 2003, Smith and Schurig carried out a comprehensive theoretical study on the fundamental principle of this exotic negative refraction [6]. The tensorial format of permittivity and permeability is used to describe the anisotropic property of the indefinite medium (the former name of HMM), where the principal components of these tensors have opposite signs. This kind of permittivity tensor leads to hyperbolic-shaped isofrequency surfaces in momentum space (k space), i.e., they are unbounded. Because the gradient of frequency in momentum space, namely, the group velocity, specifies the direction of energy flow, which is perpendicular to the isofrequency surface, negative refraction can be predicted. Experimentally, various HMMs at optical frequencies have been realized with subwavelength constituents of metal and dielectric. In this review, we first discuss the basic concepts of HMM and then review new phenomena arising from the topological transition in HMM. Next, we review the recent progress on active HMM, topologically protected edge states in HMM, and twisted HMM. Finally, we provide our prospects on future research directions of topological HMM.

## 2 HMM fundamentals

**Figure 1:**
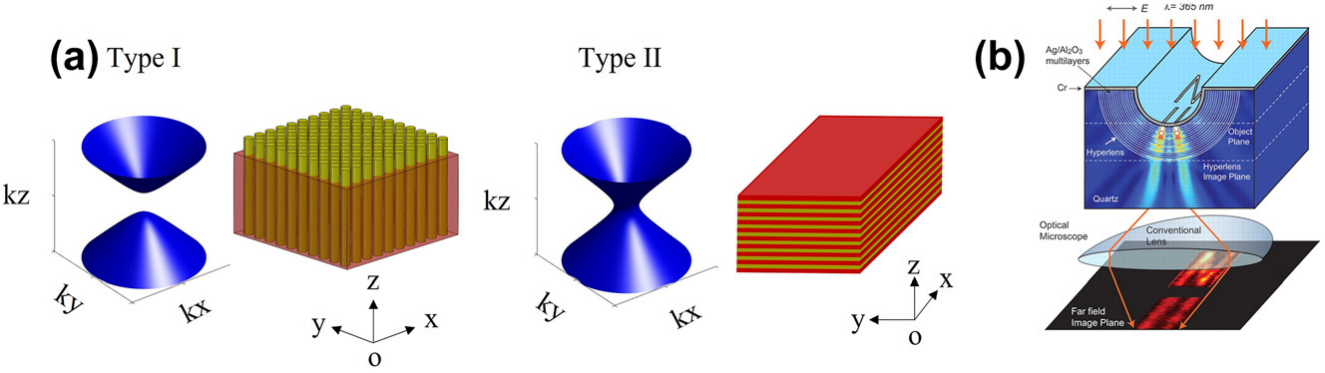
HMM fundamentals and application. (a) Isofrequency contours (TM waves) and schematic for realizing type I and type II HMM at optical frequencies. (b) Schematic of first experimental demonstration of hyperlens consisting of a curved subwavelength stack of Ag and Al_2_O_3_. Copyright 2007 AAAS [9].

The permittivity of an anisotropic medium is described in the tensorial format $\left(\begin{array}{ccc} \begin{array}{c} \varepsilon_{xx}\\ \varepsilon_{yx}\\ \varepsilon_{zx} \end{array} & \begin{array}{c} \varepsilon_{xy}\\ \varepsilon_{yy}\\ \varepsilon_{zx} \end{array} & \begin{array}{c} \varepsilon_{xz}\\ \varepsilon_{yz}\\ \varepsilon_{zz} \end{array} \end{array}\right)$ , where the principal components *ɛ*_*xx*_, *ɛ*_*yy*_, and *ɛ*_*zz*_ represent permittivities along *x*, *y*, and *z* directions, respectively [6]. For uniaxial materials, *ɛ*_*xx*_ = *ɛ*_*yy*_ ≠ *ɛ*_*zz*_, and the off-diagonal terms are all zero. In this scenario, the optical axis is along *z* direction. The dispersion relation for the medium can be derived from Maxwell’s equations [7]

$$\left(\frac{k_{x}^{2}+k_{y}^{2}}{\varepsilon_{xx}}+\frac{k_{z}^{2}}{\varepsilon_{zz}}-\frac{\omega^{2}}{c^{2}}\right)\left(\frac{k_{x}^{2}+k_{y}^{2}+k_{z}^{2}}{\varepsilon_{zz}}-\frac{\omega^{2}}{c^{2}}\right)=0,$$

where the first and second bracketed terms represent electromagnetic waves of transverse magnetic (TM) and transverse electric (TE) polarizations, respectively. When one principal permittivity component becomes negative, the isofrequency surface for the TM polarization becomes hyperbolic shape [2], [3], [4]. Figure 1 illustrates the isofrequency contours for TM waves, when *ɛ*_*zz*_ and *ɛ*_*xx*_ is negative, respectively.

In addition to anisotropy, gyromagnetic and chiral effects can also be introduced into Maxwell equations [8]. Gyromagnetic effects can be described by the off-diagonal terms in the permittivity tensor, for example, *ɛ*_*xy*_ = −*ɛ*_*xy*_. Chiral effects can be described by introducing coupling of the electrical and magnetic field in the constitutive relations

$$\overset{⇀}{D}=\varepsilon \overset{⇀}{E}+i\gamma \overset{⇀}{H}$$

$$\overset{⇀}{B}=\mu \overset{⇀}{H}-i\gamma \overset{⇀}{E}$$

where *γ* is the chirality tensor.

Usually, HMM is classified into type I (when *ɛ*_*zz*_ is negative) and type II (when *ɛ*_*xx*_ = *ɛ*_*yy*_ are negative). While there is no natural material that has a hyperbolic dispersion at optical frequencies, artificial metamaterials have allowed the realization of hyperbolic dispersion in subwavelength metal–dielectric composites, under the framework of the effective medium theory. Specifically, type I HMM is realized with metallic nanowire arrays in a dielectric surrounding, and type II HMM is realized with alternating layers of deeply subwavelength metal and dielectric multilayers. The isofrequency surfaces, as well as artificial approaches to realize type I and type II HMMs at optical frequencies, are illustrated in Figure 1(a).

The unbounded isofrequency surface can support large wave vectors; thus, HMMs can be used to realize a series of metamaterials and devices targeting applications that can benefit from overcoming the diffraction limit of light, such as super-resolution imaging and super-resolution lithography. In 2007, Liu et al. experimentally demonstrated the first hyperlens that consists of curved subwavelength stacks of Ag and Al_2_O_3_, with resolution below the diffraction limit operating at optical frequency (Figure 1(b)) [9]. Because the hyperlens has dielectric properties in the radial direction, under ultraviolet light illumination, the evanescent waves from the object can penetrate radially. On the other hand, the hyperlens has metal properties in the tangential direction. The conservation of angular momentum ensures that the tangential wave vectors are progressively compressed as the waves propagate outward, leading to the magnification of the object with super-resolution.

**Figure 2:**
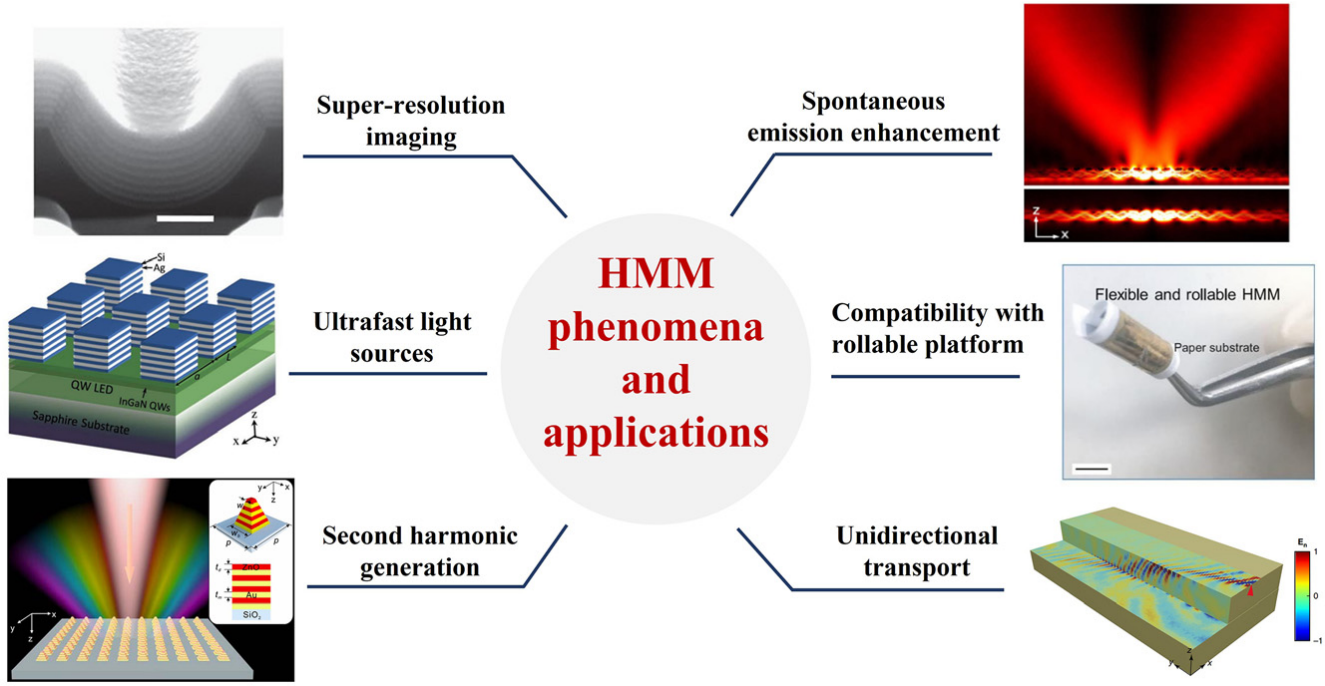
Phenomena and applications of HMM. Super-resolution imaging [15]. Copyright 2012 Springer Nature. Spontaneous emission enhancement [10]. Copyright 2015 Optica. Ultrafast light sources [16]. Copyright 2018 Wiley. Compatibility with the rollable platform [12]. Copyright 2020 Springer Nature. Second harmonic generation [13]. Copyright 2021 Springer Nature. Unidirectional transport [17]. Copyright 2017 Springer Nature.

Because an unbounded isofrequency surface can support infinite photonic density of states, another interesting phenomenon that arises in HMM is the enhancement of the spontaneous emission rate of emitters that are in close proximity of or embedded in HMM. This property makes HMM a promising platform for spontaneous emission manipulation [10] and leads to applications in ultrafast optics [11]. Since type II HMM with metal–dielectric multilayers can be easily realized with thin film deposition techniques, it is compatible with most substrates and nanostructures and is especially suitable for the rollable platform [12]. Furthermore, the strong light–matter interaction induced from subwavelength metal/dielectric features can also enhance nonlinear effects such as second harmonic generation [13]. The applications of HMM are summarized in Figure 2. At optical frequencies, in addition to the well-known super-resolution imaging, HMM can be implemented in photonic integrated circuits and photonic neural networks, which are considered to be the next generation data operation and computing platforms, respectively, with remarkable potentials due to their low energy consumption and high operation speed compared to their electronic counterpart. At terahertz (THz) frequencies, because waves can penetrate through some nonconducting materials as well as body tissues, biomedical imaging with THz wave is of great interest [14]. Thus, HMM operating at THz frequencies can be applied to increase the resolution beyond the diffraction limit. At microwave frequencies, the potential applications can be found in the future antenna design.

## 3 Dielectric-to-HMM topological transition in metamaterial

Categorized under mathematical topology, the topology and topological transition of isofrequency surface manipulates and controls the electromagnetic wave behavior, which manifests as a plethora of interesting phenomena. Consider an anisotropic dielectric medium with a permittivity tensor (*ɛ*_*XX*_, *ɛ*_*YY*_, *ɛ*_*ZZ*_) in the principal axes. If one or two of its components can evolve from positive to negative values, the isofrequency contour experiences a topological transition from ellipsoid to an unbounded hyperbolic shape [18]. Because hyperbolic dispersion can only be achieved at optical frequencies in artificially engineered materials, optical metamaterial serves as an ideal platform to study topological HMM, as one can not only engineer metamaterials to reach hyperbolic dispersion but also tune the material property across the topological transition.

**Figure 3:**
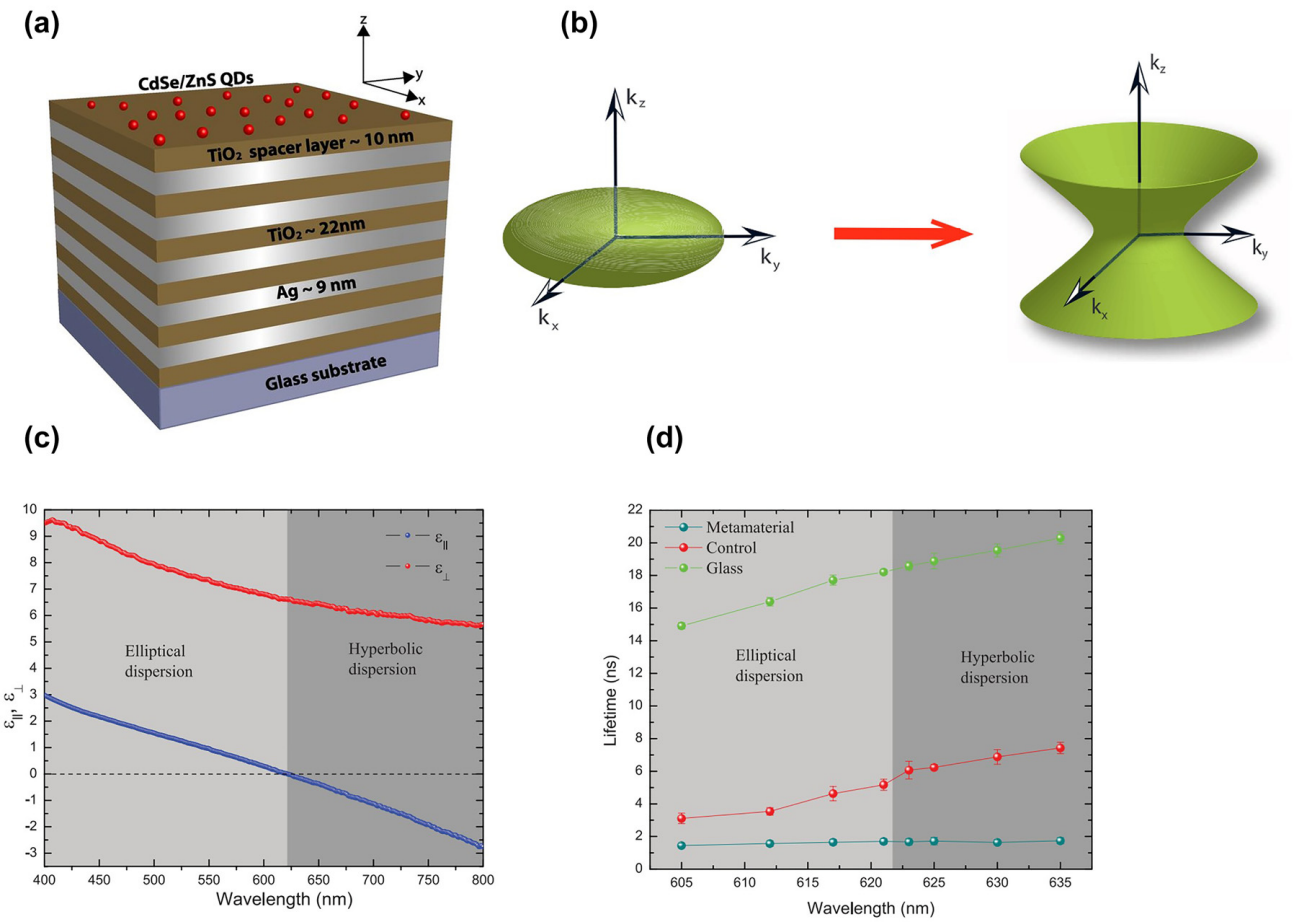
Mathematical topological transition in metamaterial consisting of TiO_2_/Ag multilayers. The isofrequency surface experiences a transition from bounded to hyperbolic shape. (a) Schematic of the HMM structure. (b) The transition of the isofrequency curve. (c) Effective permittivity for *ɛ*_‖_ and *ɛ*_⊥_. (d) Lifetime of quantum dots on metamaterials. Copyright 2012 AAAS [18].

In 2012, Krishnamoorthy et al. first experimentally demonstrated the topological transition in a TiO_2_/Ag multilayer’s isofrequency surface (Figure 3(a)) operating at visible frequency [18]. This work shows that when one of permittivity tensor’s principal component changes sign from positive to negative, achieved through wavelength tuning, the metamaterial experiences the optical equivalence of the topological Lifshitz transition. This effect is manifested by a shape change of the isofrequency surface, namely, from a bounded ellipsoidal shape to an unbounded hyperbolic shape (Figure 3(a)). Specifically, the permittivity parallel to the optical axis (*ɛ*_‖_) experiences a transition from positive to negative at 621 nm, while that perpendicular to the optical axis (*ɛ*_⊥_) remains positive (Figure 3(c)). By placing emitters (CdSe/ZnS quantum dots) close to the metamaterial surface and monitoring the spontaneous emission lifetime of these emitters at different wavelengths, the topological transition can be probed, as shown in the bottom panel of Figure 3(d).

**Figure 4:**
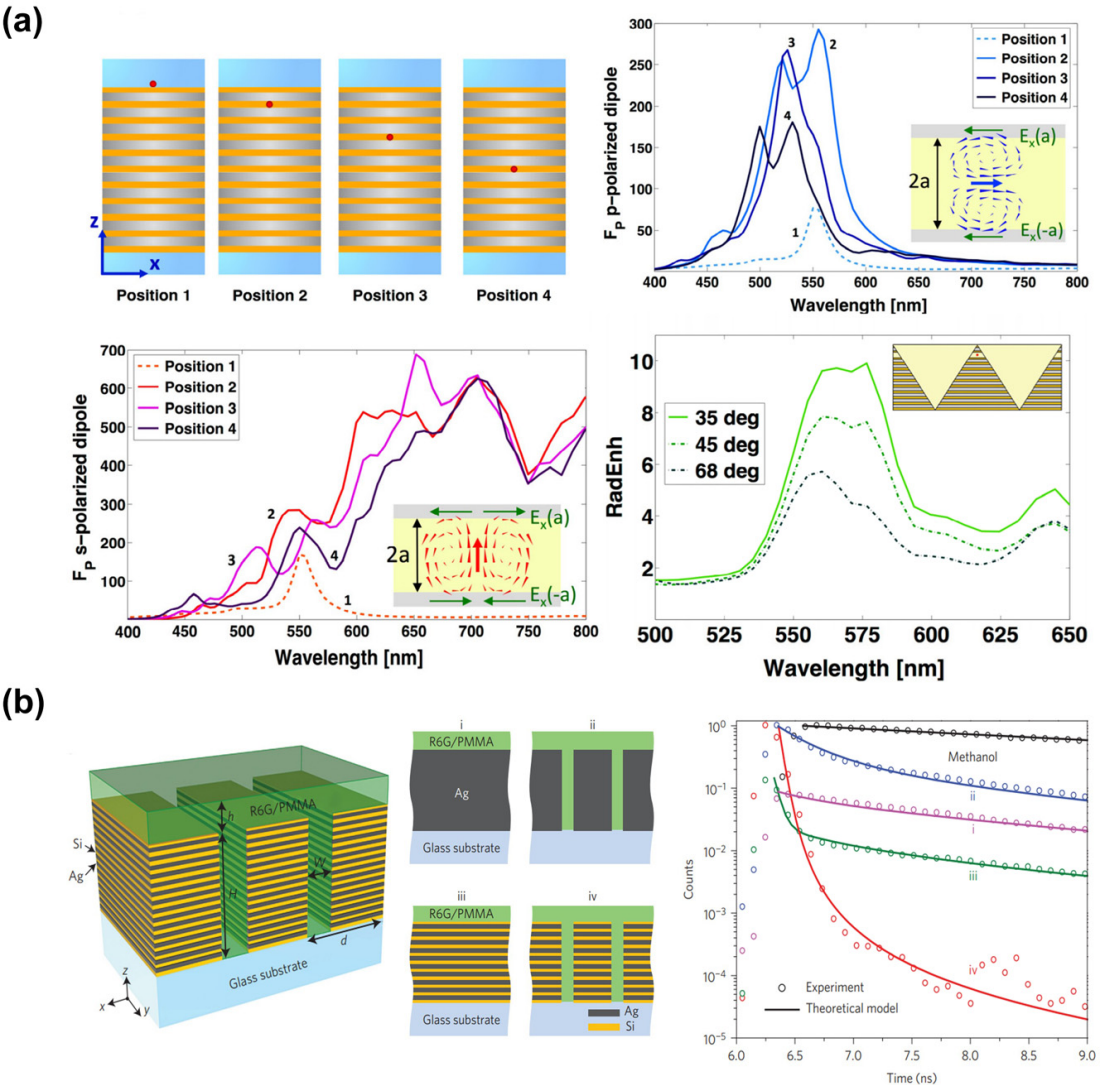
Purcell effect in HMM. (a) Theoretical study of the spontaneous emission enhancement in multilayer HMM consisting of Ag and Si multilayers [19]. Copyright 2014 Optica. (b) Experimental realization of the Purcell factor enhancement in nanopatterned Ag/Si multilayer HMM [20]. Copyright 2014 Springer Nature.

Subsequently, in 2014, Ferrari et al. investigated the Purcell effect, which describes the modification of the spontaneous emission rate in an environment compared to free space, in an Ag/Si multilayer HMM (Figure 1(b)) [19]. Using analytical modeling and full-wave electromagnetic simulation, the authors show that when an emitter (electric dipole) is placed inside the HMM structure, the spontaneous emission rate is 3 times higher than that when the emitter is placed outside the cavity (top panel of Figure 4(a)). In addition, the bandwidth of Purcell enhancement is also enlarged (lower left plot in Figure 4(a)) compared to that when the emitter is placed outside (curve 1 in the upper right plot in Figure 3(b)). Since the k vectors of the HMM can be large, a 1D grating was used to couple waves with large k vectors into free space, which leads to a 10-fold increase of the radiative emission rate (lower right plot in Figure 4(a)). The same group subsequently experimentally verified their theoretical finding using R6G dye molecules as emitters on Ag/Si multilayer HMM with sputtered Ag and Si layers (Figure 4(b)), with a nanograting out-coupler fabricated by focused ion beam milling [20]. The emission performance of the HMM with and without the nanograting out-coupler was also compared. Thanks to the momentum matching provided by the nanograting, Figure 4(b) shows that the evanescent plasmonic wave outcoupled from the HMM with nanograting is 80-fold enhanced in emission intensity compared to the HMM without nanograting. Over the next few years, spontaneous emission enhancement by HMM was also investigated in other gain media, such as 2D material [21], dye molecules [22], and InGaN multiple quantum well [16].

**Figure 5:**
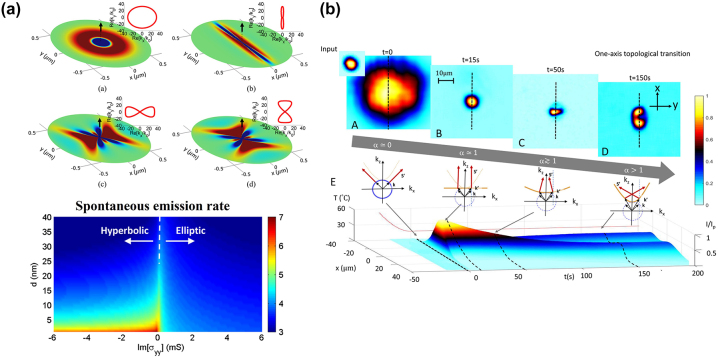
Topological transition from dielectric to HMM. (a) Topological transition in *σ*-near-zero metasurface consisting of array of densely packed graphene strips [23]. Copyright 2015 American Physical Society. (b) Topological transition induced by photorefractive nonlinear effects in KTN perovskite [24]. Copyright 2021 Springer Nature.

The topological transition in the isofrequency surface from bounded elliptical to unbounded hyperbolic shape has also been investigated in ultrathin anisotropic conductivity (*σ*)-near-zero uniaxial metasurfaces, which can be realized with an array of densely packed graphene strips operating at THz frequency [23]. Figure 5(a) shows that elliptical isofrequency surface contour appears when both the conductivity components along the surface have positive imaginary parts, and thereby the surface behaves inductively. Hyperbolic topology arises when one direction of the surface behaves as dielectric, and the other direction behaves as metal. Therefore, the topological transition occurs when one of the conductivity components exhibits a much smaller imaginary part than the other one. Due to the high Purcell factor associated with the hyperbolic dispersion, when an emitter is placed close to the surface, the spontaneous emission rate can be enhanced by several orders of magnitude in the hyperbolic regime compared to the rate in the elliptical one (bottom panel of Figure 5(a)).

The topological transition of dispersion has also been observed in a top-seeded solution grown potassium-tantalate-niobate (KTN) perovskite by manipulating nonlinear effects (Figure 5(b)) [24]. In this work, the diffusive photorefractive nonlinearity is induced by the giant electro-optic response when KTN is close to the paraelectric-to-ferroelectric phase transition condition. In this system, the dispersion relation can be written as $\left(1-\alpha^{2}\right)\left(k_{x}^{2}+k_{y}^{2}\right)+k_{z}^{2}=k_{0}^{2}n^{2}$ , where *α* is the diffusive length dominated by the quadratic electro-optic coefficient and low-frequency susceptibility. The topological transition appears when *α* = 1. The spontaneous emission rate for regions of *α* < 1 (hyperbolic), *α* ≅ 1 (hyperbolic-to-elliptic transition point), and *α* > 1 (elliptic) are depicted in the bottom panel of Figure 5(a). To observe the topological transition, a Gaussian-shaped laser beam with 46 μW power at 488 nm wavelength was first focused into a 20 μm beam waist in the plane 5 mm away from the sample surface. In so doing, the beam spot size on the sample surface is a diffracted Gaussian beam with a 50 μm beam waist. The photorefractive effect in KTN film is cumulative with respect to the sample’s exposure time optical illumination. After a time duration (time stamp *t* = 150 s in Figure 5(b)), *α* can reach steady state, which, if larger than the topological transition point, the KTN film operates in the hyperbolic regime and superlensing can be observed.

### 3.1 Gain-assisted HMM

In optical HMMs, in order to construct hyperbolic dispersion, the metallic constituent is indispensable. However, intrinsic metal loss at optical frequencies restricts access to high k-states, rendering most of HMM’s unique properties experimentally unattainable. One strategy to reduce loss in HMM is to incorporate optical gain, leading to the exploration of second-generation HMM, also termed active HMM, where gain material partially or entirely makes up the dielectric constituent. Early works disperse organic dyes into dielectrics or place transition metal dichalcogenides 2D gain material on top of HMM, and as a result, the dielectric component is only partially composed of gain [22], [25]. To maximally compensate loss, it is desirable to have the dielectric solely composed of gain.

**Figure 6:**
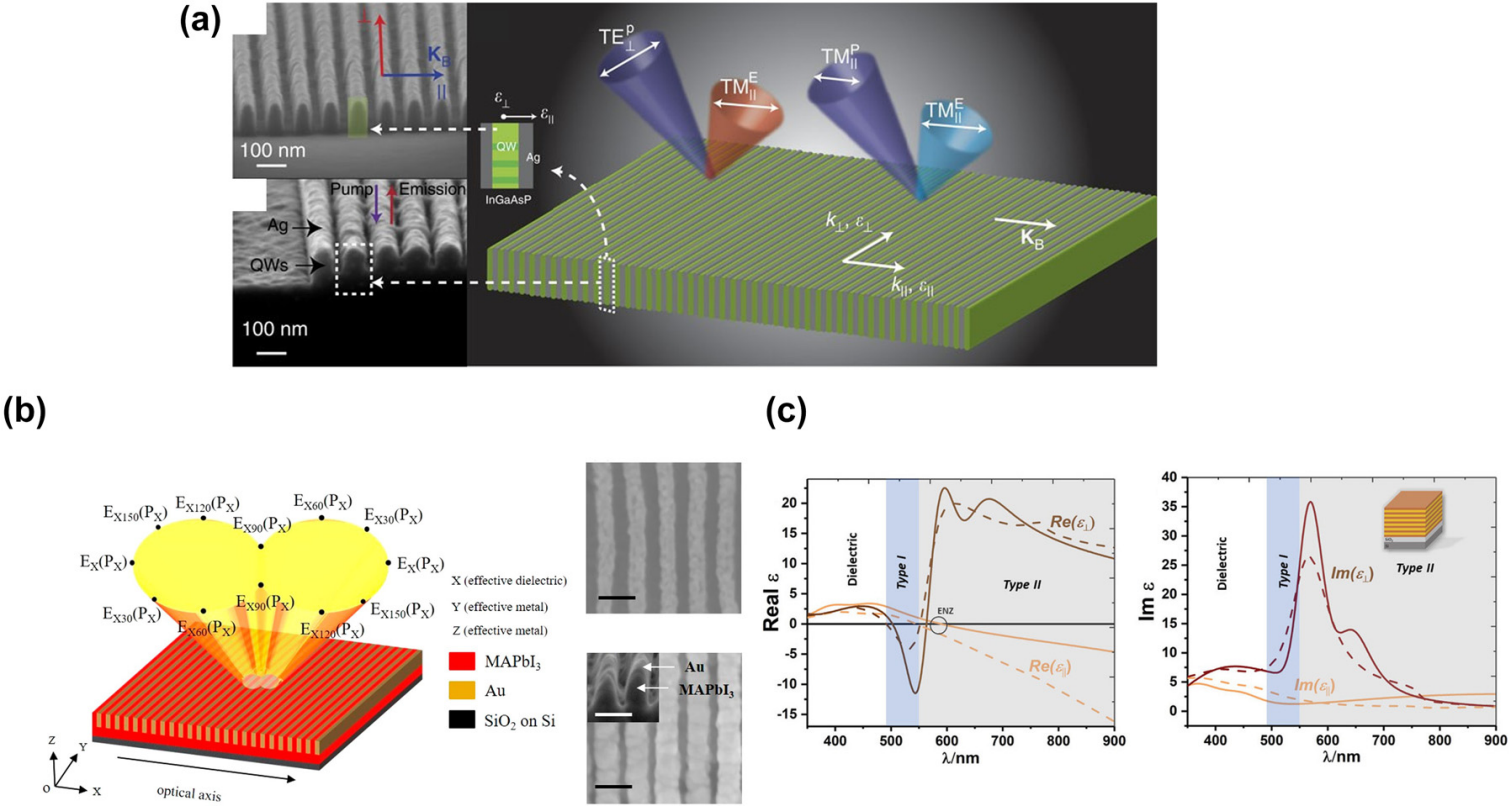
Gain-assisted HMM. (a) Luminescent InGaAsP/Au active HMM at 1550 nm [26]. Copyright 2016 Springer Nature. (b) MAPbI_3_ perovskite/Au active HMM operating at 780 nm [27]. Copyright 2020 American Chemical Society. (c) MAPbI_3_ perovskite/Au lateral HMM that can transition from type I to II [28]. Copyright 2020 Wiley.

In 2018, Smalley et al. demonstrated the first active HMM in which InGaAsP gain material completely makes up the dielectric constituent, in the telecommunication wavelength of 1550 nm (Figure 6(a)) [26]. The HMM consists of multilayers of Ag and InGaAsP, although the layers alternate in the lateral direction and has finite height. Taking advantage of the luminescent nature of InGaAsP upon optical excitation, the authors investigate the anisotropic property of HMM through polarization-dependent emission measurement. Later, similar approaches were applied to shorter wavelengths [25]. In 2020, with a similar lateral multilayer design but with perovskite/Au constituents, Li et al. realized the first active HMM below 1 μm in which the dielectric is entirely composed of gain material [27]. The sample is fabricated by thermal nanoimprint lithography to construct the MAPbI_3_ subwavelength grating, followed by the evaporation of Au to fill the air trench. In the MAPbI_3_/Au HMM, Au is chosen as the metal constituent because it doesn’t react with MAPbI_3_ and has the lowest loss among metals (top panel in Figure 6(b)). Experimentally, hyperbolicity is verified by polarization anisotropy measurements for both absorption and emission. The highest emission intensity is observed in the effective dielectric direction, while the lowest emission intensity occurs in the effective metal level. The strength of anisotropy is verified by calculating the degree of linear polarization (DOLP). For HMM samples with the same Au fraction, a smaller lattice period corresponds to a higher DOLP, indicative of the better effectiveness of the effective medium theory at more deeply subwavelength scales (bottom panel in Figure 6(b)). Compared to 3D HMM, the 2D hyperbolic metasurface configuration is more desirable and exhibits high anisotropy behavior, highlighting the integration of on-chip light sources with tailorable polarization. Subsequently 2021, Basak et al. constructed a similar layered MAPbI_3_ perovskite/Au HMM but with the traditional iteratively deposited multilayer thin films (Figure 6(c)) [28]. By tuning the MAPbI_3_ and Au thicknesses, respectively, the authors observed the transition from type I to type II.

### 3.2 Topological edge state in HMM

Although the topological transition of the isofrequency surface is categorized under mathematical topology, topological photonics phenomena stemming from the physics of topological insulators have also been found in HMM. In a condensed matter fermionic system, the remarkable property of topological phases of matter dictates that a topological insulator is conducting at the boundary but insulating in the bulk. As a consequence, the conducting edge and insulating bulk are maintained despite material defects, as long as the bulk-edge correspondence is maintained. Inspired by this, the photonic analog of topological phases of matter prompted the blooming of topological photonics in the early 2010s, providing a fascinating platform to manipulate electromagnetic waves against system disorders or defects. Compared with electronic systems, one advantage of photonics is the ease of realizing non-Hermitian effects by simply including active material as gain and metal as loss. Thus, a series of interesting non-Hermitian photonics effects have been demonstrated, such as topological lasers, topological exceptional rings [29], and bulk Fermi arcs [30]. Topological features in the photonics platform can be described by the band theory under Bloch basis, because of the analogy between Maxwell equations and the Schrödinger Equation. Different from solid-state material and photonic crystals that have translational symmetry, HMM can be treated as a non-Hermitian continuous medium under the effective medium theory approximation, which has the potential to manifest distinct topological characteristics [31].

**Figure 7:**
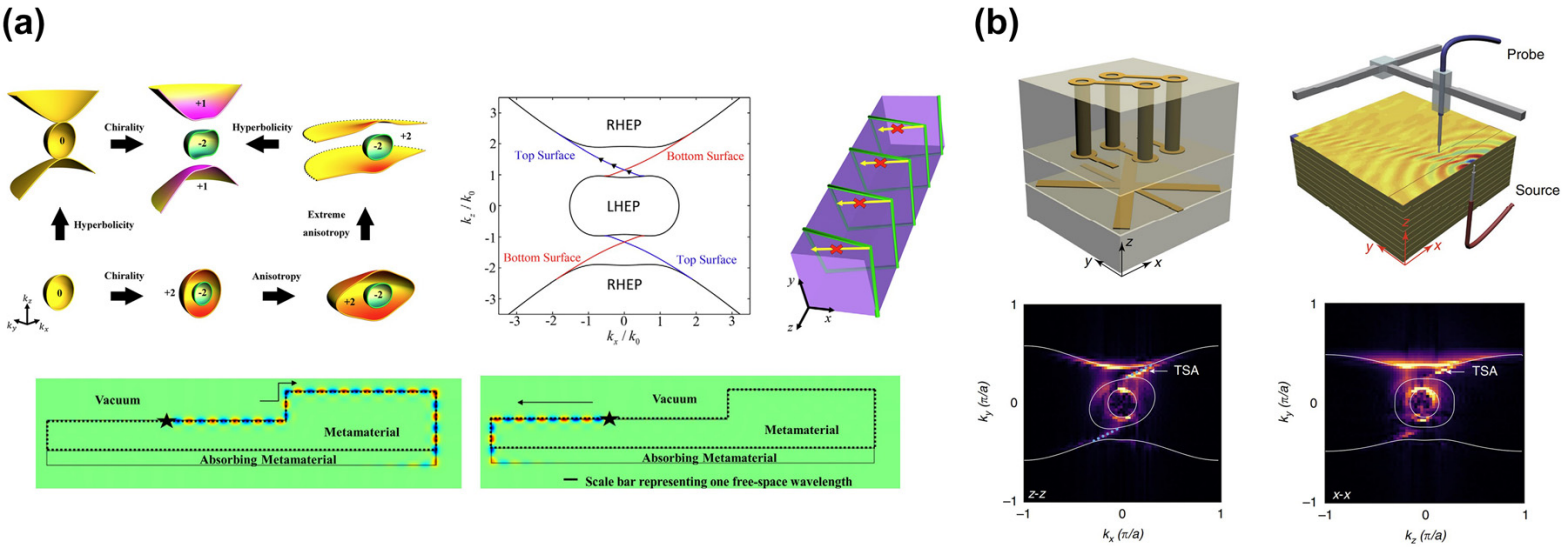
Topological edge state in HMM. (a) Topological edge state in HMM described under isofrequency surface analysis [32]. The isofrequeny can be gapped when chiral effects are incorporated. The topological surface wave can be visualized in numerical simulation when the frequency is in the surface arc. Copyright 2015 American Physical Society. (b) Experimentally observed topological edge mode in HMM in microwave frequency [17]. Copyright 2017 Springer Nature.

In 2015, Gao et al. discovered for the first time the possibility that HMM can support topological edge states [32]. By introducing chirality into HMM’s constituent materials, Figure 7(a) shows that the degeneracy of the TE and TM-like isofrequency surfaces is lifted, leading to nontrivial isofrequency surfaces with opposite nonzero Chern numbers. The surface waves at the interface between the HMM and vacuum can be solved numerically in reciprocal space, resulting in an isofrequency surface arc linking the isofrequency surface contours for TE and TM polarizations, shown in the top panel of Figure 7(a). In real space, full wave simulations can further verify the existence of topological edge states. The edge state propagates along the boundary between HMM and vacuum unidirectionally and does not scatter or reflect in the presence of the sharp corners, as shown in the bottom panel in Figure 7(a). When the HMM chirality direction is altered, the propagation direction of the surface wave is also flipped. In 2017, the same group experimentally demonstrated this phenomenon in microwave frequencies (Figure 7(b)) [17]. The chiral HMM with broken inversion symmetry is designed as a photonic type II Weyl node on a commercial printed circuit board. The structure consists of periodic stacking of trilayers of chiral, blank, and hyperbolic materials, respectively, which as a whole enables chiral hyperbolic properties (upper left plot in Figure 7(b)). The isofrequency surface is characterized by a vector network analyzer and a near-field antenna to provide the electromagnetic wave excitation (upper right plot in Figure 7(b)). The band structure of the surface state can then be obtained from the Fourier analysis of spatially distributed electric field at each frequency. The topological surface-state arcs are clearly recognized in the experiment (bottom panel in Figure 7(b)).

**Figure 8:**
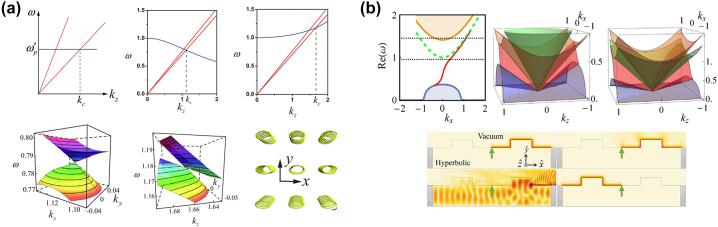
Band descritpon in topological HMM. (a) Band crossing points (type I and type II Weyl points) in chiral hyperbolic medium [35]. Copyright 2016 American Physical Society. (b) Topological edge mode described by band theory [37]. The band gap can be lifted when the gyromagnetic effects or chiral effects are incorporated into HMM. The topological surface wave can be predicted in numerical simulations. Copyright 2020 American Physical Society.

After this discovery, other types of topologically protected edge states have also been theoretically predicted in magnetized plasma [33] and in gyromagnetic material with broken duality symmetry [34]. Different types of hyperbolic dispersion have also been proposed by introducing chirality. Notably, the shape of hyperbolic dispersion is closely associated to the band crossing point dictated by the Weyl point. In 2016, Xiao et al. theoretically proposed the type I and type II Weyl points in type I HMM (the material behaves as dielectric in *x* and *y* directions and behaves like metal in *z* direction) when considering the nonlocal response of *ɛ*_*zz*_ [35], which is described by $\varepsilon_{zz}=1-\frac{\omega_{p}^{\prime 2}}{\omega^{2}}+\gamma_{zz}^{2}+\alpha k_{z}^{2}$ . Therefore, *ɛ*_*zz*_ is dependent on *k*_*z*_. The term $\frac{\omega_{p}^{\prime 2}}{\omega^{2}}$ represents Drude dispersion and *γ*_*zz*_ is the chirality along *z* direction. The permittivity along *x* and *y* directions are *ɛ*_*x*_ = 2 and *ɛ*_*y*_ = 1.7, respectively. When *α* = 0 (nonlocal effect is not included), the three modes (two transverse modes in red and one longitudinal mode in blue) are shown in the left plot in the top panel of Figure 8(a). When *α* > 0, the conventional conical-shaped bands are degenerate at type I Weyl point (middle plot in the top panel of Figure 8(a)). When *α* < 0, the conical bands are strongly tilted and degenerate at type II Weyl point (right plot in the top panel of Figure 8(a)). The corresponding bands at *k*_*y*_ and *k*_*z*_ planes are shown in the lower panel of Figure 8(a), in which the band crossing points are linear for all directions. The lower right plot of Figure 8(a) shows the proposed experimental realization of nonlocal permittivity, with arrays of elliptical helix metallic wires with helix direction along *z* direction to support chirality *γ*_*zz*_. This structure can provide a plasmonic response as well as the nonlocality. The elliptical shape of the helix structure ensures the anisotropic dielectric permittivity in the *x* and *y* directions. Other types of band-crossing features, such as nodal links, were also investigated [36].

Although the analysis and measurement of the isofrequency surface in HMM has led to many discoveries, the isofrequency surface cannot fully capture the topological features. Instead, a band description is required to fully describe the topological features of HMM. In this regard, Hou et al. developed a band theory for HMM, using a non-Hermitian Hamiltonian formulated from Maxwell’s equations [37]. Importantly, the vacuum band (green dashed line in the upper left plot of Figure 8(b)) plays a significant role in analyzing the bulk-edge correspondence in HMMs. The Hamiltonian for HMM has 6 bands solved from the eigenvalue format of Maxwell’s equations, and two types of topological edge states can be generated by either introducing chiral effects or gyromagnetic effects. Different from the electronic system, a vacuum is not an insulator for photons, so the vacuum band is required to establish the bulk-edge correspondence. Taking HMM with gyromagnetic materials as an example, when the gyromagnetic effect increases, the band gap of the HMM increases, and when the gyromagnetic effect is strong enough, the upper HMM bulk band will surpass the vacuum band. The process is depicted in the top panel of Figure 8(b). The green curve in the 2D band diagram depicts the vacuum band, while the yellow and blue curves show bands of the metamaterial. The middle and right plots are bands when the gyromagnetic effects are weak and strong, respectively. In this scenario, the topological edge state connects the lower HMM band and the vacuum band. Full wave simulation in the bottom panel of Figure 8(b) shows that, when the frequency of the excited source is below the vacuum band, the topological edge mode propagates unidirectionally along the interface between the HMM and vacuum, which can immune the reflection and back-scattering. When the source frequency overlaps the vacuum band but still lies in the band gap of the bulk HMM, the topological surface waves can still propagate along the interface but scatter at the sharp corners of the interface. However, when the source frequency has an overlap with both vacuum and bulk bands, the electromagnetic energy diffuses to both the HMM and vacuum region. Similar evolution of the band structure and topological surface waves can be observed when the chiral effects are introduced into the HMM. It is also worth noting that the band theory is developed in a continuous HMM medium approximated under the effective medium theory, which is different from the usual photonic band theory in a lattice system with a periodic index modulation. It is worth noting that this theory is applicable to all frequencies, as long as the effective medium theory is satisfied, i.e., the structure feature size is at least one order of magnitude smaller than the wavelength. Experimentally, the microwave frequency range is the preferred candidate to validate the theory because not only is metal’s intrinsic loss negligible but also easy-to-manufacture millimeter-scale structures are required.

## 4 Topological edge states in chiral media with hyperbolic isofrequency surface

Well-established methods to realize HMM at optical frequency, whether it is metal–dielectric multilayer or metal nanowires in a dielectric host, involve metal as illustrated in Figure 1. Because metal is lossy at optical frequencies, the performance of the optical devices using HMM will inevitably suffer. Although we discussed that compensating metal loss with optical gain is a viable approach, an even better approach would be to form hyperbolic isofrequency surfaces with only lossless dielectric materials. Surprisingly, this is possible with chiral dielectrics.

Optical chirality is defined by the coupling of electric and magnetic fields (commonly denoted by the term *χ* in the constitutive relations), which enables a new degree of freedom, namely, handedness, to control and manipulate electromagnetic waves at the subwavelength scale. Optical activity was first observed through circular dichroism in chiral material such as quartz and liquid crystals that are made of helical molecules [38], [39]. The artificial version of such chiral material was subsequently pursued using micro/nanostructures with broken mirror symmetry [40], [41], [42]. Thus, beams with helical wavefront carrying orbital angular momentum can be generated and have been widely investigated in a range of optical platforms such as microcavity [43] and metamaterials [44]. This has also opened a route to develop topological HMM.

**Figure 9:**
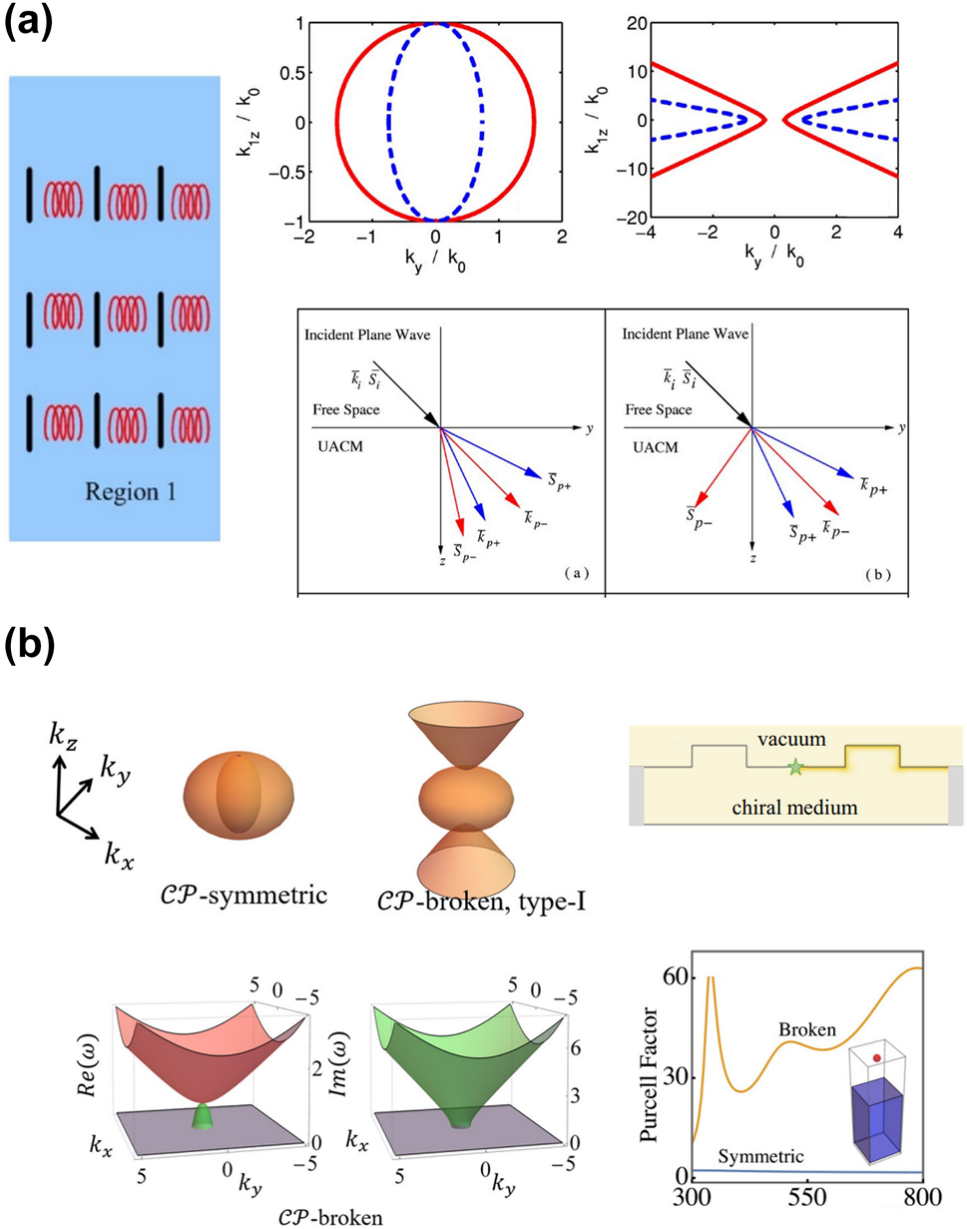
Topological edge state in chiral HMM. (a) Negative refraction in a chiral dielectric medium consisting of Swiss-roll arrays [45]. Copyright 2006 American Physical Society. (b) Hyperbolic isofrequency associated phenomenon induced by CP symmetry in a chiral dielectric medium. A large Purcell enhancement can be predicted [47]. Copyright 2021 American Physical Society.

In 2006, only a few years after the proposal of the indefinite medium, Cheng et al. theoretically demonstrated the existence of a hyperbolic isofrequency surface in a uniaxial anisotropic chiral metamaterial, in which negative refraction was also predicted. This metamaterial was realized with Swiss-roll arrays in which chirality only occurs in one direction (schematically shown in the left plot in Figure 9(a)) [45]. The *ρ* ^+^ and *ρ* ^−^ waves are the solutions of the dispersion relation obtained from Maxwell’s equations. When the chirality strength is weak, both waves (*ρ* ^+^ and *ρ* ^−^) exhibit ellipsoidal isofrequency surfaces. However, when the chirality strength is strong, the isofrequency surface for the *ρ* ^+^ wave remains elliptical in shape, while that for the *ρ* ^−^ wave exhibits a hyperbolic shape (upper right plots in Figure 9(a)). The Poynting vector and group refraction angle can also be calculated from the dispersion relation. The lower right plots in Figure 9(a) show that, in accordance with the isofrequency surface behavior, both waves have positive group velocity and group refraction angles in the case of weak chirality, but with strong chirality, the *ρ* ^−^ wave shows negative group velocity and negative group refraction. Surface waves in specific frequency ranges are also predicted at the interface between an isotropic and a biaxial anisotropic media [46].

To understand whether chiral effects can raise new properties or phenomena in HMM, Hou et al. theoretically investigated transitions in isofrequency surface and photonic band in a dielectric medium when chirality is introduced (Figure 9(b)) with symmetry considerations [47]. In short, charge-conjugation (*C*), parity (*P*), and time reversal (*T*) symmetries exist in all physical systems, and for regular dielectric, metal, and HMM, these three symmetries are always preserved individually. However, because chirality breaks the mirror symmetry, in a chiral medium, *C* and *P* symmetries break individually but preserve jointly, analogous to PT symmetry in photonics. With the chiral medium described by an isotropic dielectric permittivity tensor *ɛ*_*D*_ and anisotropic chiral tensor *γ* = diag(*γ*_*x*_, *γ*_*y*_, *γ*_*z*_), it has an exception point when $\gamma_{z}=\sqrt{\varepsilon_{D}\mu_{D}}$ . The isofrequency surfaces in Figure 9(b) show that, when *γ*_*z*_ is smaller than the value at the exceptional point, the system is in the CP-symmetric region and the isofrequency surface is bounded and elliptical. When *γ*_*z*_ is greater than that at the exceptional point, the system is in the CP-symmetry broken region and two purely imaginary eigenmodes appear, forming unbounded isofrequency surfaces, denoting hyperbolic behavior. Therefore, although the material under consideration is not a typical HMM but rather a chiral dielectric material, its hyperbolic isofrequency surface warrants its comparison with HMM. The complex band diagram of such an all-dielectric HMM is shown in the lower left plots in Figure 9(b). Topologically protected edge states can also be found at the interface between the all-dielectric HMM and air (upper right plot of Figure 9(b)). The negative refractive index observed in HMM can also be observed in this chiral dielectric. Additionally, the hyperbolic bands in the *CP*-broken regime also exhibit a large photonic density of states, and consequently, spontaneous emission enhancement if an emitting dipole is located close to the chiral dielectric medium (bottom right plot in Figure 9(b)). This work suggested a new approach toward generating unbounded hyperbolic-shaped isofrequency surface in a purely dielectric medium, in which the metal constituent is not necessary, opening a new route toward lossless HMMs.

**Figure 10:**
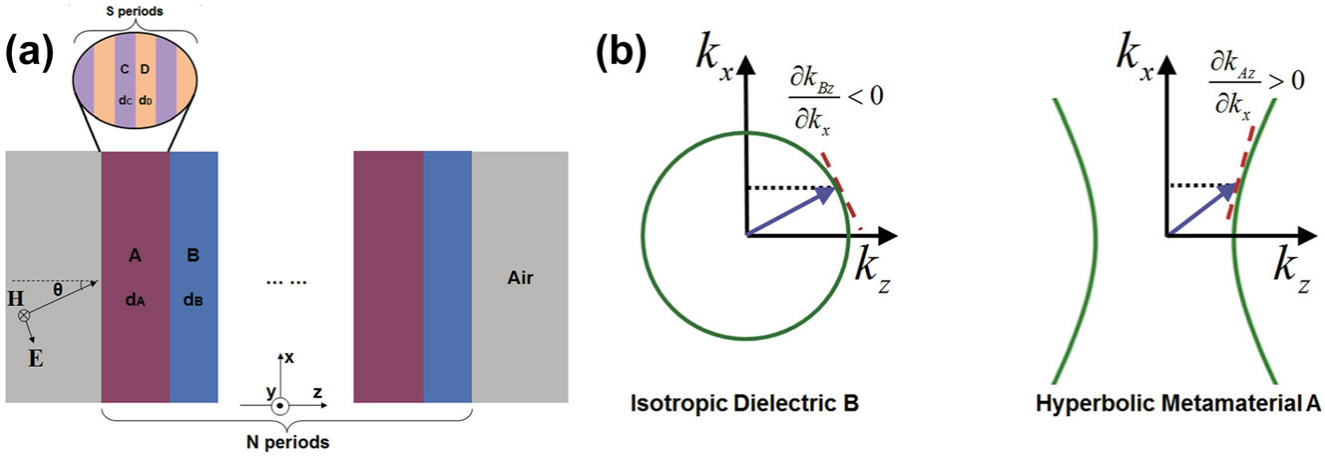
Phase variation compensation effects in HMM. (a) Alternating layers of chiral and HMM [48]. (b) Phase variation compensation effect in 1D HMM/dielectric [48]. Copyright 2016 American Physical Society.

In addition to isofrequency surfaces, photonic bands are also investigated in 1D photonic crystals containing alternating layers of HMM and regular dielectric, which support phase variation compensation (schematic in Figure 10(a)) [48]. The total propagating phase in a photonic crystal is governed by $\Phi =\left(k_{Az}d_{A}+k_{Bz}d_{B}\right)|\omega_{\mathrm{Bragg}}=\pi$ . In regular photonic crystal containing two dielectric materials, both *k*_*Az*_ and *k*_*Bz*_ decrease as the incident angle increases. Thus, *ω*_*Bragg*_ is incident angle-dependent. However, the situation is different when one of the constituent materials of the photonic crystal is HMM. As shown in the right plot in Figure 10(b), $\frac{\partial k_{Az}}{\partial k_{x}}$ and $\frac{\partial k_{Bz}}{\partial k_{X}}$ have opposite signs. The total phase variation can be compensated, and thereby it is independent of the incident angle. In the proposed realistic design, the HMM region is composed of subwavelength layered TiO_2_ and Ag with 0.5 filling factor and the dielectric region is purely TiO_2_ [49], [50]

## 5 Topology in twisted HMM

In recent years, an emerging field of research, twistronics, is rapidly growing, aiming to manipulate electron wave functions by means of rotations [51], [52]. Within this context, surprising phenomena such as superconductivity has been unveiled in bilayer 2D materials in which the two layers are rotated by the Moiré angle, also known as the magic twist angle [53]. Inspired by this principle, manipulating light–matter interactions in stacked metamaterial or 2D materials via controlling the twist angle between layers has emerged as a new research direction [54]. In this regard, when two or more layers of metasurfaces with exotic anisotropic behaviors, such as hyperbolic dispersion, interface at a twist angle, new properties can arise from the coupling and hybridization between modes in individual stacks.

**Figure 11:**
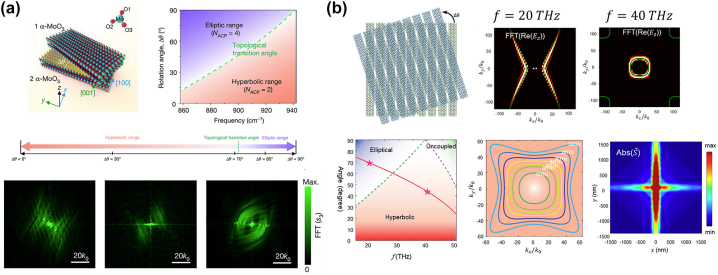
Topological transition in twisted HMM. (a) Topological transition from elliptical to hyperbolic dispersion in twisted *α*-MoO_3_ [56]. Copyright 2020 Springer Nature. (b) Moiré hyperbolic metasurface consists of twisted densely packed graphene nanoribbons [58]. Copyright 2021 American Chemical Society.

In 2019, Kotov and Lozovik theoretically proposed a generalized 4 × 4 T-matrix containing TM-TE polarization mixing to describe the optical topological transition of isofrequency surface for arbitrary bianisotropic bilayers [55]. In 2020, Hu et al. experimentally demonstrated the topological transition from hyperbolic to elliptical isofrequency contour in two stacked layers of *α*-phase molybdenum trioxide (*α*-MoO_3_) flakes (top panel in Figure 11(a)) [56], with each layer supporting in-plane hyperbolic phonon polaritons. When the two layers *α*-MoO_3_ layers are stacked with the [100] crystal direction oriented by the magic twist angle, the hyperbolic isofrequency surface of each individual layer intersects with the other forming the anticrossing points. A topological transition from hyperbolic to elliptical dispersion arises at a frequency of 925.9 cm^−1^ when the twisted angle is 180-2*β*, where *β* = atan(*ɛ*_11_/*ɛ*_22_), with *ɛ*_11_ and *ɛ*_22_ being the permittivity along [100] and [001] crystal directions, respectively. When the twisted angle is smaller than |180 − 2*β*|, the bilayer exhibits hyperbolic dispersion; when it is larger than |180 − 2*β*|, elliptical dispersion is seen. The topological feature can be described by the number of anticrossing nodes such that it is 2 in the hyperbolic region and 4 in the elliptical region. The topological transition phase diagram with respect to the twisted angle and frequency is shown in the top right plot in Figure 11(a). Experimentally, the dispersion was characterized using scattering-type scanning near-field optical microscopy (s-SNOM), a well-known scheme to characterize polaritons in the near field [57]. After the Fourier transformation of the real space image, the momentum space (k-space) dispersions were obtained. The bottom panel in Figure 11(a) shows the k-space image for −44°, 65°, and −77° twist angles, respectively. This topological transition is robust against geometrical variations such as changes in layer thickness. In the same year, the same research group further investigated the exotic topological property of twisted bilayer HMM and proposed a generalized Moiré hyperbolic metasurface (Figure 11(b)) [58]. The schematic illustration in Figure 11(b) shows twisted densely packed graphene nanoribbons, which can support in-plane hyperbolic dispersion. The dispersion relation of the system can be obtained from the poles of dyadic Green’s function for TE and TM modes in the source-free Maxwell’s equations. For Moiré HMM, the two hyperbolic dispersion branches experience anticrossing features in the isofrequency surface. When the twisted angle is 90°, it exhibits elliptical dispersion at 20 THz but hyperbolic dispersion at 40 THz (upper right plots in Figure 11(b)). The phase transition among elliptical, hyperbolic, and uncoupled are thoroughly investigated with respect to rotation angle and frequency (lower left phase diagram in Figure 11(b)). By rotating the evanescently coupled hyperbolic metasurfaces, broadband field canalization and plasmon spin-Hall phenomena can be achieved (lower-middle and -right plots in Figure 11(b)). At 90° twist angle, when frequency increases from 15 THz to 28 THz, the hyperbolic dispersion ensures that the Poynting vector and group velocity of the waves propagate in four directions, indicating canalization, which only appears in each individual HMM when absorption is strong.

**Figure 12:**
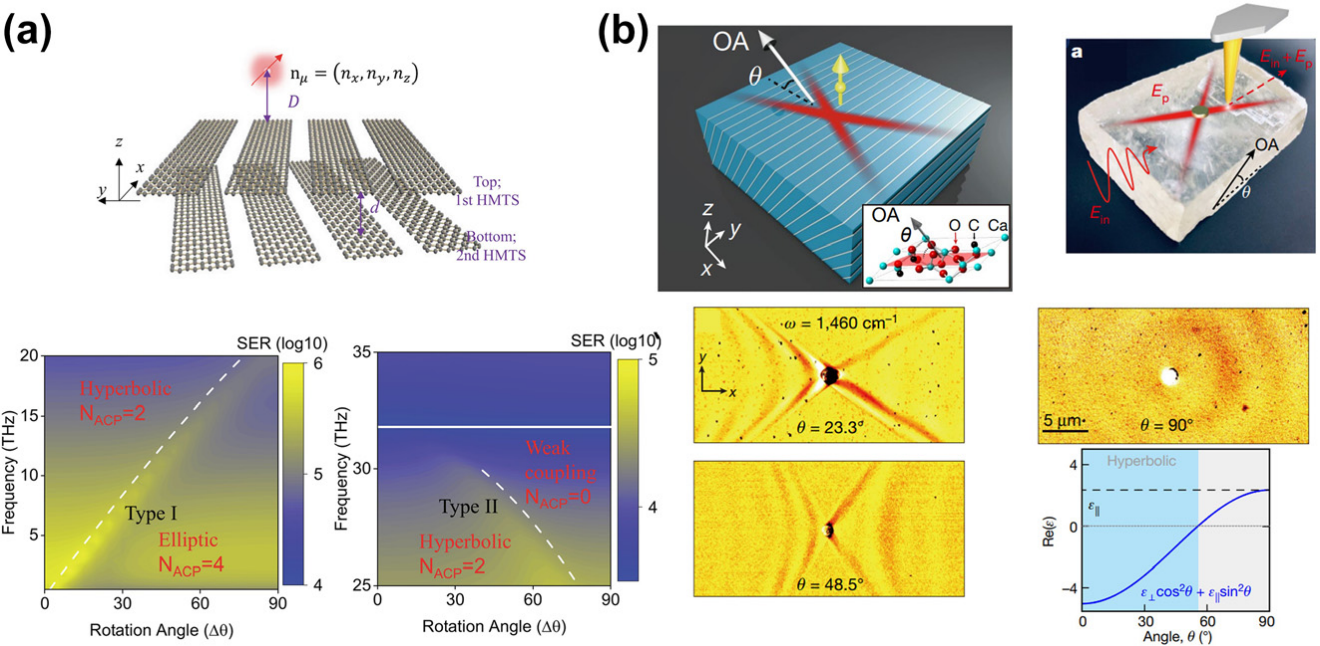
Enhancement in twisted HMM and Ghost HMM. (a) Strong light–matter interaction in twisted HMM consisting of subwavelength graphene nanoribbon array [59]. Copyright 2021 AIP Publishing Group. (d) Ghost hyperbolic polaritons in calcite crystal lattice [64]. Copyright 2021 Springer Nature.

In 2021, the same group also found the enhanced photonic density of states in twisted bilayer graphene HMM. Strong spontaneous emission enhancement rate can be achieved when the dipole emitter is placed close to the surface of HMM and operated close to the topological transition angle (Figure 12(a)) [59]. The material platform is again subwavelength graphene nanoribbon array. Figure 12(a) also shows that the hyperbolic to elliptical transition occurs at type I magic angle, while hyperbolic to weak-coupling transition occurs at type II magic angle. For spontaneous emission enhancement that is a signature of HMM, the photonic density of states of the twisted bilayer, which is proportional to the spontaneous emission enhancement, can be obtained from the dispersion calculated through dyadic Green’s function of the system. Because resonant features are indicative of strong light–matter interaction, the bottom panel in Figure 12(a) shows that a resonance appears near the magic angle for both type I and type II at THz frequency, which is not the case for a single graphene layer. Similar topological transitions in phonon polaritons have also been observed in graphene and *α*-MoO_3_ heterostructure [60], [61], [62]. Besides the bilayer HMM, topological transition in trilayer HMM has also been investigated [63].

Another atypical surface wave is the ghost polariton, which arises at the interface of a uniaxial material in which the optical axis has an angle with respect to the surface. In 2021, ghost hyperbolic surface polaritons were first revealed in calcite, an anisotropic crystal with hyperbolic dispersion in mid-infrared frequencies (Figure 12(b)) [64]. Thanks to calcite’s oblique trigonal crystal lattice, the optical axis of the crystal lattice (labeled as OA in the upper left schematic in Figure 12(b)) is tilted by angle *θ* with respect to the sample surface, leading to the emergence of ghost hyperbolic surface polaritons. Manipulation of the in-plane crystal optical axis direction relative to the surface changes the twist angle, and as such, allows tuning across the topological transition. Though first developed in bilayers, similar hyperbolic to elliptical topological transition can be achieved without the necessity of bilayers or heterostructures, by leveraging the out-of-plane optical axis. Experimentally, different twist angle *θ* can be achieved by mechanically cutting the calcite crystal along different planes. s-SNOW characterization of the dispersion relation (excited by a quantum cascade laser operating at 1310–1470 cm^−1^) shows that the polaritons propagate following a hyperbolic shape when *θ* = 23.3° and 48.5°. On the other hand, an isotropic circular polariton propagation is observed when *θ* = 90°, indicative of the elliptical region. The topological phase transition map is also outlined in the lower right plot in Figure 12(b).

## 6 Conclusion and outlook

In the last two decades, tremendous research efforts have been dedicated to HMM, and in the last decade, to the exploration of topological phenomena in HMM. Benefiting from the remarkable characteristics of topological phases of matter in both mathematics and physics, topological HMM manifests great merits in manipulating electromagnetic waves in novel ways. For example, the topological phase transition of the isofrequency surface from elliptical to hyperbolic changes the wave behavior, leading to the enhancement of spontaneous emission, negative refraction, the emergence of ghost polariton, and more. Topological protected edge states can also appear at the air and HMM interface when non-Hermitian effects such as chirality and gyromagnetism are introduced, leading to the proposal of naturally lossless all-dielectric HMM. However, the experimental realization at optical frequencies remains a challenge. One reason is that the deep-subwavelength nanostructure requirement translates to a feature size of tens of nanometers, which requires high-precision lithography, such as e-beam lithography, to define the structure. Another difficulty is the incorporation of gyromagnetic effects or chiral effects. Including gyromagnetic materials in HMM not only makes the fabrication more complicated but also requires an external magnetic field to operate. As for chiral effects, chirality is usually realized in helix structures, and fabricating such structures with a few-nanometer resolution is challenging. These difficulties will certainly also hinder optical HMM’s insertion into large-scale photonic integrated circuits. Future research may benefit by advancing the following directions: (1) experimentally achieve unbounded hyperbolic dispersion from all-dielectric chiral media. The intrinsic metal loss in conventional metal–dielectric HMM attenuates the high-k wave intensity, which hampers HMM performance. Thus far, unbounded isofrequency surface in chiral dielectric media has only been proposed theoretically, so the experimental realization will definitely be a breakthrough for the insertion of HMM in next-generation photonic devices with minimal loss. (2) Explore HMM’s application in optical processing and computing. The high-k waves supported by HMM carry information with high spatial resolution, which should bring versatility in optical processing in traditional optical computing and emerging optical neural networks. Besides, as nonlinear operation is required in many neuromorphic computing scenarios, HMM’s applications, such as spontaneous emission enhancement, lasing, and second harmonic generation, can accommodate this requirement well. Therefore, it can serve as a nonlinear component, such as a neuron, in optical neural networks. In particular, the incorporation of topological features and potential lossless realization can yield robust and stable operation against scattering and system disorders. We believe these future research directions can enrich existing research in topological HMM and suggest more approaches for HMM’s insertion in future nanophotonic devices and applications.
